# The Utility of AI in Writing a Scientific Review Article on the Impacts of COVID-19 on Musculoskeletal Health

**DOI:** 10.1007/s11914-023-00855-x

**Published:** 2024-01-13

**Authors:** Olatundun D. Awosanya, Alexander Harris, Amy Creecy, Xian Qiao, Angela J. Toepp, Thomas McCune, Melissa A. Kacena, Marie V. Ozanne

**Affiliations:** 1 Department of Orthopaedic Surgery, Indiana University School of Medicine, Indianapolis, IN USA; 2 Critical Care, and Sleep Specialists, SMG Pulmonary, Norfolk, VA USA; 3 Division of Pulmonary and Critical Care Medicine, Eastern Virginia Medical School, Norfolk, VA USA; 4 Department of Internal Medicine, Eastern Virginia Medical School, Norfolk, VA USA; 5 Sentara Health, Enterprise Analytics, Norfolk, VA USA; 6 Division of Nephrology, Eastern Virginia Medical School, Norfolk, VA USA; 7 Richard L. Roudebush VA Medical Center, Indianapolis, IN USA; 8 Department of Mathematics and Statistics, Mount Holyoke College, South Hadley, MA USA

**Keywords:** AI, ChatGPT, Scientific review article, Query, COVID-19, Bone Loss, Osteoporosis, Fracture, SARS-CoV-2

## Abstract

**Purpose of Review:**

There were two primary purposes to our reviews. First, to provide an update to the scientific community about the impacts of COVID-19 on musculoskeletal health. Second, was to determine the value of using a large language model, ChatGPT 4.0, in the process of writing a scientific review article. To accomplish these objectives, we originally set out to write three review articles on the topic using different methods to produce the initial drafts of the review articles. The first review article was written in the traditional manner by humans, the second was to be written exclusively using ChatGPT (AI-only or AIO), and the third approach was to input the outline and references selected by humans from approach 1 into ChatGPT, using the AI to assist in completing the writing (AI-assisted or AIA). All review articles were extensively fact-checked and edited by all co-authors leading to the final drafts of the manuscripts, which were significantly different from the initial drafts.

**Recent Findings:**

Unfortunately, during this process, it became clear that approach 2 was not feasible for a very recent topic like COVID-19 as at the time, ChatGPT 4.0 had a cutoff date of September 2021 and all articles published after this date had to be provided to ChatGPT, making approaches 2 and 3 virtually identical. Therefore, only two approaches and two review articles were written (human and AI-assisted). Here we found that the human-only approach took less time to complete than the AI-assisted approach. This was largely due to the number of hours required to fact-check and edit the AI-assisted manuscript. Of note, the AI-assisted approach resulted in inaccurate attributions of references (about 20%) and had a higher similarity index suggesting an increased risk of plagiarism.

**Summary:**

The main aim of this project was to determine whether the use of AI could improve the process of writing a scientific review article. Based on our experience, with the current state of technology, it would not be advised to solely use AI to write a scientific review article, especially on a recent topic.

**Supplementary Information:**

The online version contains supplementary material available at 10.1007/s11914-023-00855-x.

## Introduction

Artificial intelligence (AI) language models have been in development for years and recently there has been an exponential growth in their utilization. The application of these large language models (LLMs) has rapidly expanded into an ever-growing number and range of industries. As such, discussion concerning the application of AI in medicine and research have become a heated topic for debate [1, 2]. Indeed, use of AI requires much scrutiny and skepticism as there have been notable instances where the misuse of AI, such as ChatGPT, has led to serious consequences. With one instance resulting in the fining of two lawyers who cited fictitious court citations generated by ChatGPT [3].

Moreover, some of the greatest ethical concerns for AI consist of privacy, bias, discrimination, and the need for human judgment. Despite these concerns, the use of AI in academic research is likely unavoidable because of the potential to make processes more efficient. To test this possibility in scientific writing, we created an experiment to see if the current state of LLMs, and more specifically ChatGPT 4.0, would be able to increase the efficiency of writing scientific review articles. To accomplish this, we first identified 3 topics of importance to the musculoskeletal research field: (1) COVID-19 and musculoskeletal health [4, 5]; (2) the intersection of Alzheimer’s disease and bone [6–8]; and (3) the neural regulation of fracture healing [9–11]. We then implemented 3 approaches to write the first draft of review articles on each topic: (1) human only; (2) ChatGPT 4.0 only (AI-only or AIO); and (3) a combination of approaches 1 and 2 (AI-assisted or AIA).

The original goal was to have a total of 9 review articles and quantifiable data related to each step of the processes to assess the benefits and limitations of each approach. Please refer to the Introductory Comment [12] for more information regarding the specifics of the study design. We had several hypotheses for this experiment. First, the AI-only approach would have the highest number of inaccuracies but would take the least amount of time. Second, the human review article approach would require the least amount of change between the initial and final drafts but would be most time intensive. Third, the AI-assisted approach would take an intermediate amount of time to complete but would require fewer changes than the AI-only approach. Unfortunately, early in the process it became clear that for a very new area of investigation, like COVID-19, the knowledge cutoff date at the time for ChatGPT 4.0 of September 2021 limited its utility, especially as we found the chatbot’s tendency to fabricate information. Thus, approach 2 was abandoned for the COVID-19 topic due to significant errors and need to input most of the publications for ChatGPT, resulting in significant overlap between approaches 2 and 3. The remainder of this Comment focuses on the findings for the COVID-19 topic and we point the reader to the Comments associated with “The intersection of Alzheimer’s disease and bone [13]” and “Neural regulation of fracture healing [14]” to learn more about the specific findings of our two other studies.

## Results

A summary of time spent on various aspects of the COVID-19 and musculoskeletal health review articles completed is shown in Table 1. Since the AI-only article (approach 2) was abandoned, we did not show the full details of timing for this approach in Table 1. However, the time it took for AI fact-checking prior to abandonment was already noted to be 158% longer than that of AI-assisted approach (28.36 h). On the other hand, the total writing time was significantly less than both the human and AI-assisted approaches at 1.51 h for writing alone and 12.11 h if preparation, literature review, outlining, and writing were combined (compared to 58.5 and 77.85 h, respectively, for human and AI-assisted approaches). Of the two approaches used through completion of review articles on COVID-19 and musculoskeletal health, the AI-assisted approach required the most time to complete with a total time of 219.09 h compared to the human-only approach total time of 114.66 h.

**Table 1:** The amount of time, in hours, spent on producing various aspects for each of the two COVID-19 and musculoskeletal health review articles

Activity	Human	AI-assisted
Preparation (h)	0	13.00
Literature review (h)	45.10	45.10
Outline (h)	0.50	0.50
Writing (h)	12.90	19.25
AI fact-checking (h)	0	11.00
Student edits (h)	36.92	73.25
Faculty edits (h)	17.24	54.74
Other (h)	2.00	2.25
Total time (h)	114.66	219.09

The first drafts of the AI-only and AI-assisted manuscript were generated by ChatGPT 4.0 from queries written by the human authors. Supplementary Materials 1&2 show the complete list of queries used during these processes. Prior to abandonment, it took 67 queries to generate the first draft and 54 additional queries to make revisions for the AI-only manuscript for a total of 121 queries. This was significantly lower than the 190 queries needed to generate the first draft of the AI-assisted manuscript. Moreover, an additional 278 queries were needed to complete the edits to generate the final manuscript.

The first draft of the human, AI-only, and AI-assisted manuscripts can be observed in Supplementary Materials 3–5. The level of similarity between the initial and final drafts of the human and AI-assisted manuscripts for the COVID-19 and musculoskeletal health reviews is shown in Table 2. Ultimately, the human manuscript had a greater transformation with a 49.4% difference from the initial draft than the AI-assisted manuscript which had a 46.0% difference.

**Table 2:** The similarity between the initial and final drafts of the COVID-19 and musculoskeletal health manuscripts

Level of similarity	Human	AI-assisted
Identical (%)	9.3	5.5
Minor changes (%)	21.1	22.9
Paraphrased (%)	20.2	25.6
Different (%)	49.4	46.0

The number and accuracy of the references generated by ChatGPT 4.0 in the initial drafts of the manuscripts are shown in Table 3. Because of the importance of accuracy in scientific writing, we thought the readers would appreciate seeing the details related to the AI-only manuscript, even though the manuscript was not completed. There was a total of 113 AI-cited references in the initial draft of the AI-only manuscript. Nineteen (16.8%) AI-generated references did not exist and 31 (27.4%) references existed but were incorrect due to either incorrect authors, journal name, article title, publication year, journal volume and issue, page numbers, or DOIs. An additional 30 (26.6%) of the AI-only-cited references were misattributed to what was stated in the associated sentence (reference was legitimate but the text was not reflective of the cited reference). Therefore, a total of 80 (70.8%) AI-only-generated references were incorrect. By contrast, in the original draft of the AI-assisted manuscript, there were a total of 89 AI-cited references and 18 (20.2%) of these references were cited as incorrect due to either the reference not matching what was stated in the associated text or plagiarism.

**Table 3:** The number of AI references that were correct in the initial drafts of the COVID-19 and musculoskeletal health manuscripts

Reference criteria	AI-only	AI-assisted
AI-cited references	113	89
Correct AI-cited references	33	71
Incorrect AI references	80	18
Incorrect reference percentage	70.8%	20.2%

The similarity index generated from the plagiarism software for the initial and final drafts of the human-only and AI-assisted manuscripts is shown in Table 4. The AI-assisted manuscript had an initial similarity index of 25% and a final similarity index of 19, while the human-only manuscript had an initial similarity index of 8% and a final similarity index of 13%. It should be noted that a significant number of references were added by faculty co-authors during the revision/editing phase which likely accounts for the increase in the similarity index between the initial and final drafts for the human-only manuscript.

**Table 4:** The similarity index for the initial drafts for COVID-19 and musculoskeletal health manuscripts

Draft version similarity index (%)	Human	AI-assisted
First draft	8	25
Final draft	13	19

## Discussion

The main objective for this project was to determine whether AI could increase the efficiency in writing a scientific review article. To accomplish this, three approaches were taken to writing the first draft of scientific review articles as detailed before (e.g., human, AI-only, and AI-assisted). Of note, both of the AI approaches required humans to write the queries. However, only the human and AI-assisted articles were completed as there was too much overlap of the AI-only with AI-assisted due to the knowledge cutoff of ChatGPT.

With respect to the total time spent writing the COVID-19 and musculoskeletal health review articles, the human-only manuscript required less total time to complete at 114.66 h as compared to 219.09 h for the AI-assisted manuscript. In reviewing the data in Table 1, the higher number of hours spent during the student and faculty editing phase for the AI-assisted manuscript compared to the other two approaches may reflect the scientific writing experience of the first author. Indeed, the first author of the human-only manuscript is a senior postdoctoral fellow with several first author published manuscripts. The first author of the AI-only manuscript is a senior PhD student with 2 first author papers, whereas the first author of the AI-assisted manuscript is a medical student with only 1 previous first author manuscript. That said, it was found that ChatGPT 4.0 had a tendency to write broad, generalized statements without supporting facts and used many of the words of its limited word count on transition and concluding sentences. Additionally, even when given original research articles, ChatGPT 4.0 would frequently present the conclusion of the article but leave out details as far as the experimental design and specific results. When one paper in the section discussing the effect of SARS-CoV-2 infection on bone in animal models had differing results from the other papers, ChatGPT 4.0 could not provide a possible reason for the differing results and thus misreported the paper’s results to agree with the others. Furthermore, the chatbot was told to cite a specific article in a specific section but would not always identify and concisely summarize the relevant information from the article. For example, when prompted to cite a paper discussing various osteoporosis treatments including targeting the NLRP3 inflammasome that the human-written paper used to emphasize the potential role of the NLRP3 inflammasome in bone loss, ChatGPT 4.0 described osteoporosis and discussed treatments without specifically mentioning the NLRP3 inflammasome. Overall, the initial draft was not viewed as good scientific writing by the co-authors of the paper and had to be modified to assess the research results more critically. This was likely another reason for the increased amount of time spent on writing and editing. The readers are encouraged to examine Supplementary Material 5 for the initial draft of the AIA paper that was more reflective of the writing of ChatGPT 4.0. It remains to be seen whether the total writing and editing time would decrease for ChatGPT 4.0 for a subsequent paper once it had determined the preferences of a particular user or as the user gave more feedback and designed better queries. We did find that we received better results when we clicked like or dislike or gave specific feedback. Further, it was important to keep all of the queries in the same chat as that allowed the AI to learn from the previous responses. Having to give the feedback and fine-tune the results did add to the total time to complete the current task but may have reduced the time in the long run.

Another observation from reviewing Table 1 is the significant time spent on fact-checking the AI-only and AI-assisted manuscripts and the combined student and faculty edits were higher in the AI-assisted versus human-only approaches. Of note, the AI-only approach did not have complete student edits or any faculty edits as it was during this time that it came to light how similar approaches 2 and 3 were becoming for this topic, and it was at this point approach 2 was abandoned. With such extensive fact-checking, it was determined that the AI-only manuscript had the highest number of inaccuracies with 70.8% of references having errors including misattributions. The AI-assisted manuscript was better, with only 20.2% of the references being misattributed, but this high error rate is unacceptable in scientific writing. This large discrepancy between approaches 2 and 3 is mostly due to the AI-assisted approach using the human-assigned references and therefore, ChatGPT 4.0 was not given the opportunity to fabricate references. However, this did not prevent instances of plagiarism and misattribution. Moreover, when we subjected the initial manuscript drafts to plagiarism detection software, the AI-assisted manuscript had a similarity index of 25% which was much higher than the human manuscript which was only 8%, suggesting a higher probability for plagiarism in the AI-assisted manuscript. This may be due to the inherent methodology of the AI-assisted approach which consisted of querying ChatGPT 4.0 to give summations of the articles to generate the manuscript. Of interest, the similarity index increased to 13% for the final draft of the human-only approach and decreased to 19% for the final draft of the AI-assisted approach. The former likely reflects the numerous edits from other co-authors focused around the addition of specific new articles or ideas. The latter may reflect the numerous edits from the other co-authors addressing and reducing the incidence of AI plagiarism.

One of the greatest hinderances faced when writing the AI-only review article was the knowledge cutoff date of September 2021, for ChatGPT 4.0 at the time we used it. This made it especially difficult to write a well-informed manuscript. Indeed, most of the literature on the topic was after this date, limiting the utility of ChatGPT in writing the manuscript without significant human assistance (e.g., providing references). Therefore, when considering use of ChatGPT for any topic, it would first be important to determine whether there is an established pool of knowledge on the topic before the knowledge cutoff date or whether this limitation has been eliminated.

There were some interesting discoveries noted while completing the initial fact-checks of the AI-only manuscript prior to its being abandoned that are worth detailing for the interested reader. Many of the references generated by the AI were claimed to be published in 2021 and 2022. However, when fact-checked, a majority of the citations the chatbot claimed to be published in 2021 were in fact not published in 2021 and some of the references were published as far back as 2008 (prior to the COVID-19 pandemic). The articles ChatGPT claimed to be published in 2022 were easily identified as either incorrectly cited or not existing as it was past the knowledge cutoff date. Due to this occurrence, we speculate that ChatGPT may be aware of its own knowledge cutoff and falsely cited the year of publication for these references to possibly compensate for this limitation.

ChatGPT had a propensity to fabricate information, creating a system where misinformation is presented as fact and misleading the user into believing the information provided is in fact true. The act of fabrication of information by AI has been termed as a “hallucination” or “artificial hallucination” [15, 16]. Hence, it has been established that in order to responsibly use ChatGPT for writing any piece of literature one must be critical in fact-checking the information synthesized from the AI. Even in a task as simple as requesting a list of reference to support an idea, it is imperative to validate that the given citations exist, are correct, and are actually relevant to the idea they are meant to support.

Generating the AI-assisted manuscript proved a host of challenges that were unique from the AI-only manuscript. The author for this review had little previous experience with using ChatGPT and consulted with a colleague with more experience for assistance through the writing process. The open-access version of ChatGPT, GPT-3.5, cannot read PDFs. Thus, a limitation to writing this manuscript was having to pay for the premium, GPT-4 version of the language model. Only through this paid subscription were the authors able to access the plugin “AskYourPDF,” which generated a unique ID for each PDF. This process required the author to categorize each code with the appropriate PDF and was a time-consuming task. The author found that when more sources were uploaded, GPT-4 became less reliable when citing where the information had been pulled from. Therefore, a limit of 8–10 articles per subsection was set as a way to mitigate this issue. During the process of editing the AI-assisted manuscript, there were multiple instances of the AI plagiarizing the title of articles in the summaries in an attempt to make it seem like a newly synthesized idea. Moreover, there were instances of the AI plagiarizing partial sentences from within the same articles, but joined them together in perhaps an attempt to avoid detection. When asked to decrease the word count of a previously generated section while preserving sources, the AI had a tendency to inappropriately group citations following a sentence, introducing another source of error.

Ultimately, with the current limitation of AI, we argue it is not possible to write an accurate, well-informed, critical scientific review solely with ChatGPT. Indeed, due to concerns such as plagiarism, depth of content, and artificial hallucinations, it would not be advised. Despite these concerns, utilizing AI as an assistant when writing scientific reviews may be possible with caveats. Perhaps the most important caveat would be to combine AI writing with strict human oversight. However, while there is no guarantee that using AI would make the overall process faster, it could make parts of the writing process faster. For example, when prompted to create an outline when initially writing the AI-only paper, the initial outline generated was deemed acceptable with minor revisions. ChatGPT could also be used as a source to overcome writer’s block and may be particularly useful to those for which English is not their native language. Importantly, ChatGPT 4.0 contains a growing number of plugins that are able to streamline the process of writing a review from reading PDFs for literature reviews to providing summaries of reference material. Thus, the expansion of these capabilities could lead to a future where the need for extensive human intervention is more limited.

### Supplementary Information

Below is the link to the electronic supplementary material.

Supplementary file1 (DOCX 1009 KB)

## Data Availability

Data available upon reasonable request.

## Declarations

### Disclaimer

The views in this article represent those of the authors and are not the official position of the agencies.
